# Does sarcopenia predict change in mobility after hip fracture? a multicenter observational study with one-year follow-up

**DOI:** 10.1186/s12877-018-0755-x

**Published:** 2018-03-05

**Authors:** Ole Martin Steihaug, Clara Gram Gjesdal, Bård Bogen, Målfrid Holen Kristoffersen, Gunhild Lien, Karl Ove Hufthammer, Anette Hylen Ranhoff

**Affiliations:** 1 Kavli Research Centre for Geriatrics and Dementia, Haraldsplass Deaconess Hospital, Bergen, Norway; 2 Department of Clinical Science, University of Bergen, Bergen, Norway; 3 Department of Rheumatology, Haukeland University Hospital, Bergen, Norway; 4 Western Norway University of Applied Sciences, Bergen, Norway; 5 Department of Orthopedics, Haukeland University Hospital, Bergen, Norway; 6 Department of Rheumatology, Diakonhjemmet Hospital, Oslo, Norway; 7 Centre for Clinical Research, Haukeland University Hospital, Bergen, Norway; 8 Department of Clinical Science, the Faculty of Medicine and Dentistry, University of Bergen, Postbox 7804, n-5020 Bergen, Norway

**Keywords:** Activities of daily living, Hip fractures, Independent living, Mobility limitation, Skilled nursing facilities, Sarcopenia

## Abstract

**Background:**

Patients with hip fracture frequently have sarcopenia and are at great risk of loss of mobility. We have investigated if sarcopenia predicts change in mobility after hip fracture.

**Methods:**

This is a prospective, multicenter observational study with one-year follow-up. Patients with hip fracture who were community-living and capable of walking before the fracture were included at three hospitals in Norway (2011–2013). The primary outcome of the study was change in mobility, measured by the New Mobility Score (NMS). Sarcopenia was determined postoperatively by anthropometry, grip strength, and NMS.

**Results:**

We included 282 participants and sarcopenia status was determined in 201, of whom 38% (77/201) had sarcopenia, 66% (128/194) had low muscle mass, 52% (116/222) had low grip strength and 8% (20/244) had low pre-fracture mobility (NMS < 5). Sarcopenia did not predict change in mobility (effect 0.2 points; 95% CI –0.5 to 0.9, *P* = 0.6), but it was associated with having lower mobility at one-year (NMS 5.8 (SD 2.3) vs. 6.8 (SD 2.2), *P* = 0.003), becoming a resident of a nursing home (odds ratio 3.2, 95% CI 0.9 to 12.4, *P* = 0.048), and the combined endpoint of becoming a resident of a skilled nursing home or death (odds ratio 3.6, 95% CI 1.2 to 12.2, *P* = 0.02).

**Conclusions:**

Sarcopenia did not predict change in mobility in the year after hip fracture.

## Background

A hip fracture is associated with severe and persisting mobility impairment in more than half of patients [1]. For the last 30 years, a substantial effort has been made to understand the condition of sarcopenia, and several definitions have been proposed [2]. Sarcopenia has recently been recognized as an independent condition with its own ICD-10 code [3]. One of the most widely used definitions is by the European Working Group on Sarcopenia in Older Persons (EWGSOP): low muscle mass with low muscle strength or low physical performance [4]. Previous studies on sarcopenia in patients with hip fracture have been cross-sectional, single-center, have included few participants or have had short follow-ups [5–10]. The three components of EWGSOP sarcopenia have different associations with mobility after hip fracture. Physical performance and mobility are strong determinants of mobility after hip fracture [11, 12]. Muscle strength is a somewhat weaker predictor [13, 14], whereas the studies on muscle mass have been inconclusive [15]. Our primary hypothesis is that sarcopenia, determined by methods suitable for bed-side use, predicts change in mobility in the year after hip fracture and therefore that sarcopenia status is useful for determining prognosis and is a possible cause of mobility impairment. Further, we aim to describe the associations of sarcopenia and the individual components of sarcopenia (muscle mass, grip strength and mobility) and adverse clinical outcomes in the year after hip fracture: change in activities of daily living, reoperations for hip fracture, all-cause hospitalization, fractures, becoming a resident of a nursing home or death.

## Methods

### Study design

We conducted a prospective observational study of sarcopenia in patients with acute hip fracture with follow-up at three months and one year, conducted at three Norwegian hospitals in 2011–2013.

### Participants

Participants were included while in hospital in the postoperative phase. Eligible participants were aged ≥65 years, able to give informed consent as judged by experienced clinicians, were living in the community, and were ambulatory before the fracture. Patients who were unstable such as with delirium, acute respiratory failure or in severe pain were not eligible. Other exclusion criteria were dementia when it made informed consent impossible, remaining life expectancy of less than three months and bone disease other than osteoporosis or osteomalacia. We screened for participants by examining lists of patients admitted for hip fracture or staying on the hospital wards.

### Data collection

Information was collected by the authors and study personnel by examination, chart review, routine blood tests and by interviews with patients and their caregivers from the first postoperative day and until discharge from hospital. Weight was measured with the scales on the hospital wards. We collected the American Society of Anesthesiologists (ASA) score, Charlson comorbidity index [16], Barthel activities of daily living (B-ADL) score [17], length of the acute care hospital stay, previous hip fracture and type of hip fracture. Follow-up was at 3 months at an outpatient clinic and at one year as a telephone interview with the patient or care-giver. Information on previous and subsequent hip fractures, and reoperations for the index hip fracture came from the Norwegian Hip Fracture Register [18]. This register started data collection in 2005 and has coverage on an estimated 90% of all hip fractures in Norway. The register has information on reoperations, with an estimated coverage of 65% of hip fractures treated with surgical pinning, 68% after hemiarthroplasty and 93% after total hip replacement [19]. Mortality data was supplied by the National Population Register, which is complete.

### Sarcopenia

Participants were classified as sarcopenic if they had low muscle mass and either low grip strength or impaired mobility, as described by the EWGSOP [4]. Total body muscle mass was determined by anthropometry by the method of Heymsfield et al. using height, arm circumference and triceps skinfold [20]. Arm circumference was measured on the right arm using a non-elastic tape at the mid-point of the acromion and olecranon process, and triceps skinfold was measured on the posterior aspect of the same arm at the same level using a skinfold caliper (Harpenden, Baty International, Great Britain). Height was measured by a wall mounted stadiometer, or if the patients was unable to stand self-reported height was used. If the participant was unable to stand or report their height, the length from heel to crown was measured while lying in bed. In cases with missing value on height at baseline, height measured at follow-up was used. The values for total body muscle mass were transformed to appendicular lean mass (ALM) using model 1 described by Kim et al. [21]. The cut-points for low muscle mass were ALM ≤7.25 kg/m^2^ for men and ≤5.67 kg/m^2^ for women. We chose anthropometry for its ease of use at the bed-side in immobile hip fracture patients. Grip strength was measured with a Jamar Hydraulic Dynamometer (Sammons Preston, USA) while the patient was sitting in bed or on a chair with the elbow flexed, the wrist in the neutral position and with verbal encouragement. Grip strength was measured three times on each hand with short intervals between each attempt while the grip was repositioned. The single best value out of these six measurements was used. Low grip strength was ≤30 kg for men and ≤20 kg for women. Mobility in the two weeks before the hip fracture was determined by interview using the New Mobility Score (NMS). The NMS is scored 0–9 according to a person’s ability to walk indoors, outdoors, or while shopping [22]. The cut-point for low mobility was chosen as < 5, as this has been used to predict mortality after hip fracture [23]. We used a Danish version of the NMS with minimal modifications to Norwegian. Sarcopenia status was determined postoperatively and at follow-up.

### Outcome measures

The primary outcome was change in mobility, calculated as NMS at one year minus the pre-fracture NMS. We believe that change in mobility is more relevant than mobility for identifying patients who are more likely to benefit from interventions. We determined mobility pre-fracture, at three months, and at one-year. All other analyses were considered exploratory. Other outcome variables at one year were NMS at one year, B-ADL at one year, change in B-ADL, new clinical fractures, new hip fractures, reoperation for hip fracture, all-cause hospitalizations, death, becoming a permanent resident of a skilled nursing home, and the combined endpoint of becoming a permanent resident of a nursing home or death. The combined endpoint was chosen because death and becoming a resident of a nursing home are competing risks. New clinical fracture was any symptomatic skeletal fracture reported by the patient.

### Statistical analysis

We report descriptive data as means with standard deviations or as counts with percentages. To examine the predictive effect of sarcopenia status on changes in mobility and level of activity of daily living, we used linear regression analyses with NMS and B-ADL as response variables and sarcopenia status at baseline (sarcopenic vs. not sarcopenic) and age, sex and BMI as predictors. Age, sex and BMI were included in the models because they are established predictors of mobility after hip fracture [24] or sarcopenia [25]. The relationship with age and BMI was not assumed to be linear and was modelled using restricted cubic splines with 3 knots, placed at the 10%, 50% and 90% quantiles. We assumed that a one-point change in NMS would be clinically significant. We used Fisher’s exact test for the analysis of sarcopenia associated with new clinical fracture, new hip fracture, reoperations, all-cause hospitalization, becoming a resident of skilled nursing home, and the combined endpoint of nursing home or death. The association between the separate components of sarcopenia (muscle mass, grip strength and mobility) with change in mobility, change in B-ADL and the combined endpoint of becoming a resident of a skilled nursing home or death was analysed using regression analysis. Muscle mass, grip strength and mobility were independent continuous variables, and were analyzed separately. Change in mobility and change in B-ADL were continuous, dependent variables and the combined endpoint of becoming resident of a nursing home or death was a dichotomous dependent variable.

For the regression analyses, we used multiple imputation (500 imputations), based on predictive mean matching, using the ‘aregImpute()’ function in the ‘rms’ R package [26]. The variables used in the imputation models were the ones included in the regression models, variables highly correlated with these variables and variables expected to explain the missing data mechanism: NMS at baseline, follow-up, and at one year, and change in NMS from baseline to one year, B-ADL before the hip fracture and change in B-ADL from pre-fracture until one year, sarcopenia status at baseline, BMI at baseline, ASA score during hip fracture surgery, previous hip fracture, serum albumin when in hospital, grip strength at follow-up, sex, clinical fractures and hip fractures in the year after admission, becoming a resident of a skilled nursing home, or dying in the following year. All continuous predictors were modelled linearly in the imputation model. The imputation analyses were done in R 3.3.0 [27] and the rest of the analyses were done in Stata 14 (Stata Corp., USA). *P*-values ≤0.05 were considered significant.

## Results

**Fig. 1:**
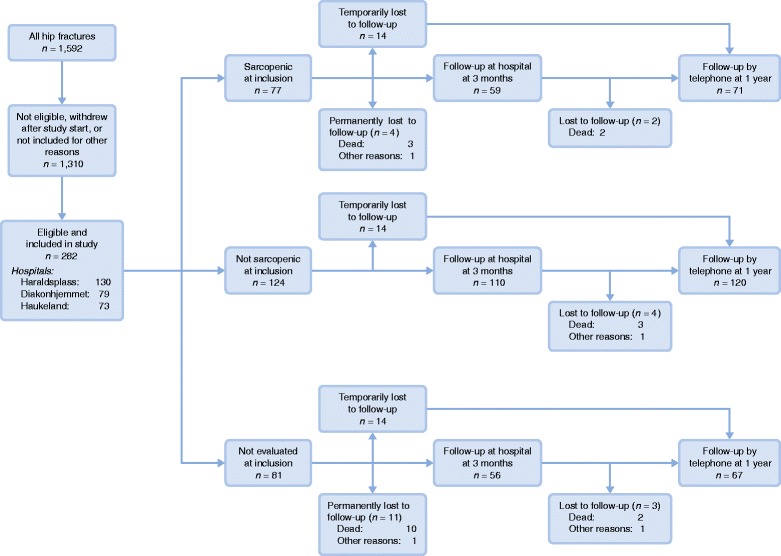
Overview of patients with hip fracture, patients included in the study and patients returning for follow-up

All patients in hospital with confirmed hip fracture were considered for inclusion if the research staffs at the different hospitals were present. Some patients were unable to participate because they were discharged before the two-day consent process was completed. There was no systematic recording of the patients who were screened, but not included. Figure 1 describes the progress of participants through hip fracture, inclusion in the study and follow-up. During the period of inclusion 1592 patients had surgery for hip fracture and 282 patients were included in the study.

Mean age was 79.4 (SD 8.2) years and 76% were female. Mean BMI was 24.1 (SD 4.3) kg/m^2^, with a wide range 13.0 to 44.7 kg/m ^2^. See Table 1 for baseline demographics. One patient died during the hospital stay. Participants who had missing data on sarcopenia status during hospitalization had lower pre-fracture NMS and pre-fracture B-ADL and were more likely to become a permanent resident of a skilled nursing home. For 69 participants, height was not assessed during the hospital stay, and for 52 of these, height determined at follow-up was used.

**Table 1:** Baseline characteristics of participants by sarcopenia status

	Not sarcopenic			Sarcopenic			*P*-value
Age, years (SD)	77.1	(7.8)	*n* = 124	81.8	(7.6)	*n* = *7*7	< 0.0001
Female, n (%)	95	(77)	*n* = 124	56	(72)	*n* = *7*7	0.5
Barthel ADL pre-fracture (SD)	19.5	(1.1)	*n* = 85	18.7	(1.9)	*n* = 60	0.006
Type of hip fracture							0.6
Neck of femur, not displaced, n (%)	29	(24)	*n* = 123	14	(18)	*n* = 77	
Neck of femur, displaced, n (%)	46	(37)	*n* = 123	31	(40)	*n* = 77	
Trochanteric, n (%)	48	(39)	*n* = 123	32	(42)	*n* = 77	
ASA score (SD)	2.3	(0.6)	*n* = 124	2.7	(0.6)	*n* = *7*7	< 0.001
Previous hip fracture, n (%)	5	(4)	*n* = 124	9	(12)	*n* = *7*7	0.039
Charlson score (SD)	0.9	(1.3)	*n* = 124	1.1	(1.3)	*n* = *7*7	0.15
Heart failure, n (%)	7	(6)	*n* = 124	6	(8)	*n* = *7*7	0.5
Previous myocardial infarction, n (%)	14	(11)	*n* = 124	9	(12)	*n* = *7*7	0.9
Cerebrovascular disease, n (%)	13	(10)	*n* = 124	8	(10)	*n* = *7*7	0.98
Diabetes mellitus, n (%)	9	(7)	*n* = 124	10	(13)	*n* = *7*7	0.2
Any solid tumor, n (%)	7	(6)	*n* = 124	8	(10)	*n* = *7*7	0.2
Pulmonary disease, n (%)	15	(12)	*n* = 124	18	(23)	*n* = *7*7	0.036
Length of hospital stay, days (SD)	6.8	(2.7)	*n* = 124	9.6	(6.7)	*n* = *7*7	< 0.001
Body composition							
BMI, kg/m^2^ (SD)	25.6	(4.2)	*n* = 107	22.1	(3.7)	*n* = 70	< 0.001
ALM/height^2^, kg/m^2^ (SD)	6.3	(1.5)	*n* = 111	4.4	(1.0)	*n* = 77	< 0.001
Women	6.1	(1.3)	*n* = 86	4.3	(0.8)	*n* = 56	< 0.001
Men	7.0	(1.7)	*n* = 25	4.8	(1.2)	*n* = 21	< 0.001
Grip strength							
Grip strength, kg (SD)	27.0	(10.3)	*n* = 123	16.5	(6.4)	*n* = 77	< 0.001
Women	22.9	(6.9)	*n* = 94	14.3	(5.0)	*n* = 56	< 0.001
Men	40.1	(8.3)	*n* = 29	22.3	(5.9)	*n* = 21	< 0.001
Mobility							
New Mobility Score (SD)	8.0	(1.5)	*n* = 123	7.1	(2.0)	*n* = 74	< 0.001
Women	8.0	(1.6)	*n* = 94	7.1	(2.0)	*n* = 55	0.008
Men	8.2	(1.4)	*n* = 29	6.8	(2.2)	*n* = 19	0.017

Baseline characteristics by sarcopenia status (means with standard deviations and counts with percentages). *P*-values for comparison of groups are by the Mann–Whitney–Wilcoxon test, except for type of fracture which is by chi-squared test. Trochanteric fractures include basocervical femoral neck fractures and subtrochanteric fractures. Previous hip fracture indicates a previous hip fracture, either left or right hip. ALM: Appendicular lean mass, ADL: Activities of daily living

### Sarcopenia

**Fig. 2:**
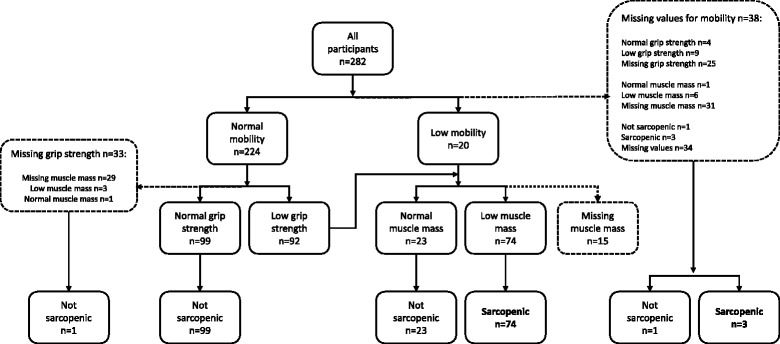
What participants were assessed for muscle mass, grip strength, mobility and sarcopenia

Sarcopenia status during hospitalization for hip fracture was determined in 201 participants, and 39% (77/201) had sarcopenia. Low muscle mass was present in 66% (128/194) of the participants, low grip strength in 52% (116/222), and 8% had low pre-fracture mobility (19/243). One participant did not have muscle mass determined but had grip strength and NMS above the cut-points and was considered not-sarcopenic. Figure 2. Participants with sarcopenia were older, had lower BMI, greater ASA score at operation, greater prevalence of previous hip fracture and pulmonary disease and lower B-ADL before the fracture. Grip strength and ALM were assessed at a median of 4 days after surgery (interquartile range 3 to 6 days) (Fig. 2).

### Outcomes after one year

**Fig. 3:**
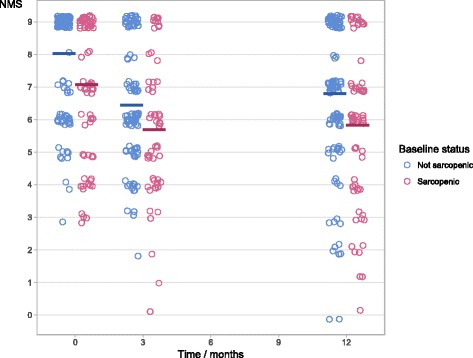
New Mobility Score (NMS) during hospitalization, at three months, and at one year, stratified by sarcopenia status during hospitalization. The horizontal lines show mean NMS scores

**Fig. 4:**
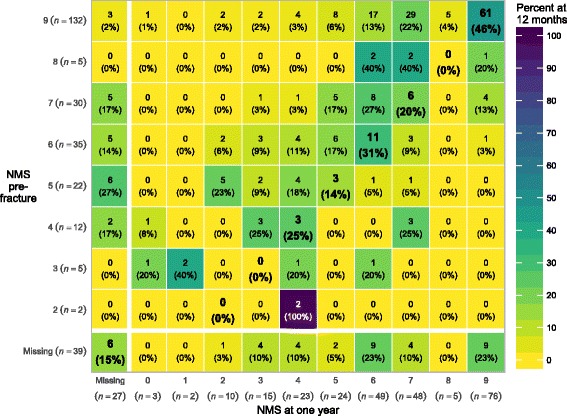
New Mobility Score (NMS) pre-fracture and at one-year follow-up. The first number in each cell is the number of patients with the given combination of NMS scores. For each row, the percentage values and the cell shadings show the distribution of NMS at follow-up for a given NMS score at baseline. No patients had a NMS of 1 at baseline, and patients with a NMS score of 0 was excluded from the study. Patients with the same NMS score at baseline and follow-up are shown in boldface, and any cell to the right of this diagonal indicates an improvement in the NMS

Sarcopenia was not associated with change in mobility at one year in unadjusted or adjusted analyses (e.g., the change in NMS was an additional 0.2 in sarcopenic patients compared to non-sarcopenic patients, 95% CI: –0.5 to 0.9, *P* = 0.6); see Table 2 for outcomes at one-year. Sarcopenia status at hospitalization did not predict change in mobility from pre-fracture to 3 months, or from 3 months to one-year. Results were not affected by imputation of missing values. Mobility was reduced in 54% of participants one year after hip fracture, with a mean NMS of 6.4 (SD 2.2). See Fig. 3 for NMS by sarcopenia status during the year after hip fracture. Figure 4 describes the relationship between specific scores on the NMS pre-fracture and at one-year. Participants with sarcopenia had lower mobility at one-year, NMS 5.8 (SD 2.3) vs. 6.8 (SD 2.2), *P* = 0.003, and greater impairment in B-ADL, 16.8 (SD 4.4) vs. 18.6 (SD 2.8), *P* = 0.001, compared to patients without sarcopenia. Sarcopenia was associated with becoming a permanent resident of a skilled nursing home (OR 3.2, 95% CI: 0.9 to 12.4, *P* = 0.048) and the combined endpoint of becoming a resident of a skilled nursing home or death (OR 3.6, 95% CI: 1.2 to 12.3, *P* = 0.02).

**Table 2:** Outcomes after one year by sarcopenia status

	Marginal values						Regression (sarcopenic – not sarcopenic)					
	Not sarcopenic			Sarcopenic			Unadjusted			Adjusted		
	Mean	(SD)	No.	Mean	(SD)	No.	Estimate	95% CI	*P*	Estimate	95% CI	*P*
Change NMS	−1.2	(1.8)	*n* = 117	−1.3	(1.9)	*n* = 67	0.0	−0.6 to 0.6	0.9	0.2	−0.5 to 0.9	0.6
Change B-ADL	−0.8	(2.4)	*n* = 76	−2.2	(3.9)	*n* = 52	−0.4	−1.2 to 0.3	0.3	−0.3	− 1.1 to 0.6	0.6
				Not Sarcopenic			Sarcopenic			OR	95% CI	*P*
Fracture, n (%) N				7	(6)	*n* = 120	8	(11)	*n* = 71	2.0	(0.6 to 7.0)	0.3
Hip fractures, n (%) N				3	(2)	*n* = 124	3	(4)	*n* = 77	1.6	(0.2 to 12.5)	0.7
Reoperations, n (%) N				7	(6)	*n* = 124	1	(1)	*n* = 77	0.2	(0.0 to 1.8)	0.2
Hospitalization, n (%) N				41	(33)	*n* = 123	24	(33)	*n* = 73	1.0	(0.5 to 1.9)	1.0
Nursing home, n (%) N				5	(4)	*n* = 124	9	(12)	*n* = 77	3.2	(0.9 to 12.4)	0.048
Death, n (%) N				3	(2)	*n* = 124	5	(6)	*n* = 77	2.8	(0.5 to 18.5)	0.3
Death or nursing home, n (%) N				6	(5)	*n* = 124	12	(16)	*n* = 77	3.6	(1.2 to 12.3)	0.02

Outcomes after one year by sarcopenia status (sarcopenic – not sarcopenic). Regression analysis for change in mobility and Barthel ADL from pre-fracture until one year adjusted for age, sex and BMI with imputation of missing values. ADL: Barthel Activities of daily living. Analysis for fracture, hip fracture, reoperations, all-cause hospitalization, nursing home, death or nursing home or death by two-sided Fisher’s exact test using available cases. NMS: New mobility score. B-ADL: Barthel activities of daily living. OR: Odds ratio

#### Muscle mass, grip strength and mobility

Muscle mass or grip strength was not associated with any outcome in adjusted analysis Table 3. In unadjusted analysis, grip strength and NMS were associated with a reduced risk of becoming a resident of a nursing home or death. The NMS was positively associated with change in B-ADL in adjusted analysis (estimate 0.2 per point, 95% CI 0.0 to 0.4, *P* = 0.03).

**Table 3:** Outcomes after one year predicted by muscle mass, grip strength or mobility

	Unadjusted β (95% Confidence interval)				Adjusted β (95% Confidence interval)			
Change in New Mobility Score at one year								
ALM/height^2^, kg/m^2^	0.0	(−0.1 to 0.2)	*p* = 0.7	*n* = 175	0.2	(−0.1 to 0.4)	*p* = 0.2	*n* = 155
Grip strength, kg	0.0	(−0.0 to 0.0)	*p* = 0.7	*n* = 193	−0.0	(−0.0 to 0.0)	*p* = 0.7	*n* = 169
Change in Barthel activities of daily living at one year								
ALM/height^2^, kg/m^2^	0.0	(−0.2 to 0.3)	*p* = 0.8	*n* = 121	0.1	(−0.2 to 0.4)	*p* = 0.4	*n* = 115
Grip strength, kg	0.0	(−0.0 to 0.1)	*p* = 0.07	*n* = 137	0.0	(0.0 to 0.1)	*p* = 0.1	*n* = 128
New Mobility Score, point	0.2	(0.0 to 0.4)	*p* = 0.03	*n* = 148	0.2	(0.0 to 0.4)	*p* = 0.03	*n* = 130
Death or nursing home at one year								
ALM/height^2^, kg/m^2^	0.8	(0.6 to 1.2)	*p* = 0.3	*n* = 194	1.0	(0.6 to 1.7)	*p* = 1.0	*n* = 170
Grip strength, kg	0.9	(0.9 to 1.0)	*p* = 0.002	*n* = 222	0.9	(0.9 to 1.0)	*p* = 0.1	*n* = 186
New Mobility Score, point	0.7	(0.6 to 0.9)	*p* < 0.001	*n* = 243	0.8	(0.6 to 1.0)	*p* = 0.06	*n* = 194

Outcomes after one year by muscle mass, grip strength or mobility. Analysis of change in mobility and Barthel activities of daily living by regression and with imputation of missing values. ALM/height^2^, grip strength and New Mobility Score are continuous, independent variables. Change in New Mobility Score and Barthel activities of daily living are continuous dependent variables. n: number of cases without missing values. OR: Odds ratio, ALM: Appendicular lean mass. Adjusted analysis with age, sex and BMI as covariates

## Discussion

The aim of this study was to investigate if sarcopenia predicted change in mobility after hip fracture. We found that sarcopenia status did not predict change in mobility in unadjusted analysis, which indicates that sarcopenia is not useful in determining prognosis. Further, sarcopenia did not predict change in mobility in analysis adjusted for age, sex and BMI, which indicates that sarcopenia status is not likely to be causally related to developing reduced mobility. We used multiple imputation to reduce the loss of information associated with missing values. This approach is considered inferior to having all the data, but preferable to performing analysis on complete data. One assumption of multiple imputation is that missing values can be estimated by the remaining information in the dataset. The results of our analysis were similar when analyzing complete cases and when analyzing datasets with imputed values, indicating that the results of our analysis are valid even if this assumption was erroneous.

Change in mobility was not associated with sarcopenia and this was consistent across all the investigated time periods, from baseline to three months, from baseline to one year and from three months to one year. Mobility from before the hip fracture until one year is characterized by an initial loss of mobility and a subsequent partial recovery. Sarcopenia is not associated with either the loss of mobility or the recovery, which further supports that sarcopenia is not related to change in mobility. In contrast to change in mobility, being sarcopenic was associated with having lower mobility pre-fracture, at three months and at one year, compared to not being sarcopenic. This is expected, since low mobility is one criteria for sarcopenia. As seen in Fig. 4, pre-fracture mobility is a determinant of mobility at three months and one year. Savino et al. found that grip strength measured in hospital predicted recovery of walking ability in patients with hip fracture [13]. In contrast, our findings indicate that neither muscle mass nor grip strength, when analysed as continuous variables, were associated with change in mobility. This indicates that the choice of cut-points for low muscle mass or low grip strength would not have changed our results. We found an association between mobility pre-fracture and change in activities in daily living, but this was an exploratory analysis and the effect size was small.

Sarcopenia was associated with an increased probability of becoming a resident of a skilled nursing home (OR 3.2, 95% CI 0.9 to 12.4, *P* = 0.048) and the combined endpoint of becoming a resident of a nursing home or death (OR 3.6, 95% CI 1.2 to 12.3, *P* = 0.02). This is a clinically relevant finding but must be interpreted with caution, as it was an exploratory outcome and we were not able to correct for age, sex or BMI because of the low number of outcomes. Among the participants who had sarcopenia status determined, 6 participants died or became permanent residents of a nursing home among the not sarcopenic and 12 participants among those who were sarcopenic. The NMS was chosen as our measure of physical performance because we assumed that many participants would be unable to walk at inclusion. The NMS is extensively studied as a predictor of mobility, morbidity, mortality and becoming a resident of a nursing home [28–31]. We found a ceiling effect with the NMS, with 54% of participants scoring the maximum 9 before the fracture and 30% at one-year. Possibly because participants with a pre-fracture NMS of 0 or 1 were not eligible for inclusion. Patients found the NMS easy to understand and scoring was straightforward. Surprisingly, we found that 8% of patients had better mobility at one-year compared to pre-fracture. For some of the patients this was due to illness that started before the fracture, and their improvement in mobility after hip fracture was due to resolution of their illness, rather than successful rehabilitation. Use of rehabilitation services improves mobility after hip fracture [32, 33]. We did not record what rehabilitation services the participants received, and it is possible that rehabilitation could mediate the effect between sarcopenia and change in mobility.

The participants in our study were slightly younger (79.4 vs. 80.0 years) and had a lower mean ASA score (2.5 vs. 2.7) indicating better health compared to patients in the Norwegian Hip Fracture Register. We did not include patients from skilled nursing homes or with severe cognitive impairment, and our results are not generalizable to those populations.

Anthropometry is considered a less valid method for determining muscle mass compared to dual-energy X-ray absorptiometry (DXA) or computed tomography scan [34]. The EWGSOP recommends not using anthropometry to determine muscle mass in research but allows for it in clinical practice [4]. We have previously investigated how anthropometry compares to DXA in identifying low muscle mass and found an area under the curve of 0.64 (95% CI 0.54–0.75) in women and 0.72 (95% CI 0.56–0.87) in men [35]. Using anthropometry to identify low muscle mass instead of DXA can lead to misclassification of muscle mass status and hence sarcopenia status. By using anthropometry to determine sarcopenia status we reduced our ability to detect an effect of sarcopenia on outcomes. We used anthropometry in our study because it is in common use [36], inexpensive, and more easily performed on patients with reduced mobility and acute illness, compared to DXA [37]. Some consider objectively measured physical performance superior to self-reported mobility, such as the NMS, but when the two types of measurement are compared in hip fracture patients they have been found to be equally predictive of outcomes [38]. Future research on sarcopenia in hip fracture patients could explore other methods for determining sarcopenia, such as computed tomography to directly measure intramuscular adipose tissue [34] or using objective measures of physical performance such as the Short Physical Performance Battery [39]. A randomized controlled study of an intervention targeting sarcopenia status to improve mobility after hip fracture would provide additional insight on the causal relation between sarcopenia and mobility.

The included patients were a minority of all patients operated on for hip fracture during the period of inclusion. We included postoperative patients who were frequently bed-bound, receiving opiates for pain relief, with indwelling urinary catheters and while receiving intravenous fluid therapy. We believe there were three main reasons for the low recruitment rate: patients did not fulfill the inclusion criteria, patients were discharged before the consent process could be completed, and participants declined to participate because it was too much of a burden. For the patients who did consent to participate we found that determining sarcopenia by anthropometry, grip strength and the NMS was feasible. The greatest difficulty was in determining the height of the participants.

## Conclusion

Sarcopenia status determined in postoperative hip fracture patients by anthropometry, grip strength and self-reported mobility did not predict change in mobility in the year after hip fracture. Sarcopenia was associated with having lower mobility at one year and a greater risk of becoming a resident of a nursing home or death.

## Abbreviations

ALM: Appendicular lean mass
ASA: ASA Physical Status Classification System score.
B-ADL: Barthel activities of daily living
BMI: Body mass index
CI: Confidence interval
DXA: Dual-energy X-ray absorptiometry
EWGSOP: European Working Group on Sarcopenia in Older Persons
NMS: New mobility score
OR: Odds ratio
SD: Standard deviation
